# “It’s about how you take in things with your brain” - young people’s perspectives on mental health and help seeking: an interview study

**DOI:** 10.1186/s12889-024-18617-4

**Published:** 2024-04-20

**Authors:** Mikael B. Andersén, Åsa Revenäs, Petra V. Lostelius, Erik M. G. Olsson, Annika Bring, Lena Ring

**Affiliations:** 1 Department of Women’s and Children’s Health, Uppsala University, Uppsala, Sweden; 2 Region Uppsala, Primary Care and Health, Uppsala, Sweden; 3 Centre for Clinical Research, Region Västmanland – Uppsala University, Västmanland Hospital Västerås, Västerås, Sweden; 4 School of Health, Care and Social Welfare, Mälardalen University, Västerås, Sweden; 5 Orthopedic clinic, Västmanland Hospital Västerås, Västerås, Sweden; 6 Clinic for Pain Rehabilitation Västmanland, Region Västmanland, Västerås, Sweden

**Keywords:** Adolescents, Young people, Youth health clinic, Youth-friendly health service, Mental health, Qualitative content analysis

## Abstract

**Introduction:**

Poor mental health in young people has become a growing problem globally over the past decades. However, young people have also been shown to underutilize available healthcare resources. The World Health Organisation (WHO) has formulated guidelines for youth-friendly health services (YFHSs) to increase youth participation in healthcare. Still, little is known about how young people using these services perceive mental health, indicating a knowledge gap concerning the subjective evaluation of their mental health.

**Aim:**

To investigate how young people visiting youth health clinics (YHC) perceive the concept of mental health and factors they view as central to maintaining mental health.

**Methods:**

In total 21 interviews were carried out, 16 in 2018, and 5 in 2023 to assure no changes in findings after the COVID-19 pandemic. Subjects were recruited during visits to youth health clinics (YHCs) in mid-Sweden and were aged 15–23 years. Recruitment strived to achieve heterogeneity in the sample concerning gender, sexual orientation, gender identity and age. Interviews were transcribed and analysed using qualitative content analysis.

**Findings:**

Findings of the analysis revealed two themes, “Mental health is helped and hindered by the surroundings” and “Mental health is difficult to understand and difficult to achieve”. The participants described their health as highly dependent on their social surroundings, and that these are important to maintaining health but may also affect health negatively. They described mixed experiences of the health care services and mentioned prerequisites for seeking care for mental health problems such as accessibility and respect for their integrity, including the right to turn down offered treatment. The informants also viewed mental health as an ongoing undertaking that one must work for, and that it is sometimes difficult to know what constitutes mental health. They also expressed a need from healthcare services to enquire about their health, and to show an active interest in how they are doing.

**Conclusions:**

Findings underline the need of young people’s individual needs to be met in the healthcare system and their vulnerability to their social surroundings. Health status assessments in young people should consider social and individual factors to fully capture mental health.

**Supplementary Information:**

The online version contains supplementary material available at 10.1186/s12889-024-18617-4.

## Introduction

Adolescence and young adulthood is a pivotal time of development in a young person’s life [1, 2]. During this period, young people go through major biological and cognitive changes as well as forming their identities both within themselves and in their social surroundings [3], and studies show that young people are particularly susceptible to social stimuli as well as prone to risk taking [4]. Although young people are generally healthy, the past decades have seen an increase in mental health problems in this age group [1, 5, 6]. It has also been shown that mental health issues often start during adolescence and young adulthood and then persist through the lifespan [7, 8]. Studies have shown increased incidence of clinical diagnoses such as depression and anxiety [9–11], as well as sub-clinical symptoms e.g. stress [12]. This trend in increasing mental health problems is particularly worrying, since studies have also found that poor mental health during childhood and adolescence is a predictor of negative outcomes in adulthood, such as poor educational attainment, unemployment, and future mental health problems [13, 14]. In contrast to this, young people also tend to underutilize available traditional healthcare services, since they are perceived as not meeting young people’s specific needs, such as a feeling of trust and mutual respect with care staff, high levels of confidentiality and help managing the perceived stigma of poor mental health [15–17]. This entails a risk of them not receiving adequate treatment for their mental health problems.

To be able to cater to the needs of adolescents and young adults, the World Health Organization (WHO) established guidelines for youth-friendly health services (YFHS) in 2012 [18], sometimes also referred to as adolescent-friendly health services. These guidelines may be formulated as eight standards [19] that in turn may be summarised in five domains: YFHSs are accessible, acceptable, equitable, appropriate, and effective [18, 20]. YFHSs may provide all types of care but are often tasked predominantly with providing care and advice for sexual health as well as mental health, but the focus of different clinics and service providers differ. The guidelines for YHFSs have been applied to health services to varying degrees in different nations [21, 22], but often on a limited project basis. Few countries have well-established structures for YFHS in place permanently.

In Sweden, there has been a system of youth health clinics (YHC) in place since the 1970’s. These clinics mostly seem to meet WHOs criteria for YFHSs [20, 23], although some domains, e.g. accessibility, vary between clinics. The clinics provide free sexual and mental health care for patients aged 12–25 years and are present in all regions of Sweden [24]. Young people may seek out the YHCs specifically for help with mental health issues, but it is also common that young people initially contact the YHCs primarily seeking other services, foremost for sexual health issues. While at the clinic other health issues e.g. poor mental health might be identified [24]. The YHCs have a stated aim of low-threshold, preventative care [24]). Studies have also found that young people using the YHCs perceive them as youth-friendly and welcoming [25], although other studies have found that young people from a non-Swedish cultural background find these clinics less accessible and less suited to their needs [26].

YHCs can play a crucial role in early detection and treatment of poor mental health, by providing accessible and available care as well as ensuring confidentiality, privacy, being non-judgmental and having an inclusive approach [18]. To ensure that adolescent voices are at the centre of decision-making processes for their mental health it is important to understand what mental health, both good and poor, means to them [27]. Some studies have explored how young people perceive health in general [27–30], with findings including the important role played by responsible adults surrounding young people [27]. Findings also show the central importance of mental health in relation to young people’s perceptions of their overall health, with physical health described in a study of 15-years old boys as “subordinate” [29]. Studies have also explored young people’s perceptions of mental health specifically [31], but no such studies have, to our knowledge, focused on patients at YFHCs specifically, and it cannot be ruled out that young people using the YHCs have unique health perceptions or healthcare needs. The aim of the present study was to investigate how young people visiting YHCs perceive the concept of mental health and factors they view as central to maintaining mental health.

## Method

### Study design

This exploratory study has an inductive qualitative content analysis design according to Graneheim and Lundman [32, 33]. The study is approved by the Swedish Ethical Review Authority, registration number 2021–04440. According to Swedish law, parental consent is only needed for children under 15 years of age [34].

### Settings and participants

Participants for the interviews were recruited during visits to six different YHCs in three different counties in central Sweden. Inclusion criteria were being at least 15 years of age and attending an appointment at one of the YHCs where recruitment took place. Exclusion criteria were not being able to understand written and spoken Swedish. Participants were aged between 15 and 22 years of age, and included 16 people identifying as female, 3 people identifying as male and 2 with other gender identities (see Table 1 for details).

**Table 1:** Description of participant characteristics

IP	Age	Place of birth	Parents place of birth	Gender identity	Sexual orientation
**1**	17	Sweden	Sweden	Female	Heterosexual
**2**	22	Sweden	Sweden & Europe	Other	Bisexual
**3**	17	Sweden	Sweden	Female	Homosexual
**4**	17	Sweden	Sweden	Female	Heterosexual
**5**	21	Sweden	Sweden	Female	Heterosexual
**6**	18	Sweden	Sweden	Female	Heterosexual
**7**	15	Europe	Europe	Other	Pansexual
**8**	18	Sweden	Sweden & OE	Female	Heterosexual
**9**	19	Sweden	Sweden	Female	Heterosexual
**10**	22	Sweden	Sweden	Male	Bisexual
**11**	19	Sweden	Sweden	Female	Heterosexual
**12**	18	Sweden	Sweden & Europe	Female	Heterosexual
**13**	22	Sweden	OE	Male	Heterosexual
**14**	21	Sweden	Sweden	Female	Heterosexual
**15**	19	Sweden	OE	Male	Heterosexual
**16**	17	OE	Sweden	Female	Heterosexual
**17**	20	Sweden	Sweden	Female	Bisexual
**18**	17	Sweden	Sweden	Female	Heterosexual
**19**	15	Sweden	Sweden	Female	Heterosexual
**20**	15	Europe	Europe	Female	Heterosexual
**21**	19	Sweden	Sweden	Female	Don’t know/un-certain

*Abbreviations:* *IP* Interview Participant, *OE* Outside Europe

Participants also included people from different backgrounds. 10 participants reported cohabiting with both parents, 5 participants lived with one parent and the remaining 6 lived either with partners, siblings, in foster care or alone. Participants had different levels of education, with the youngest participants still in secondary school, while some of the older participants were at university. Participating YHCs were located in cities, towns and in rural locations, which was also reflected in participants having both urban and rural backgrounds. Purposive sampling using a stratified approach was used [35] in order to recruit a heterogeneous group of participants concerning gender, gender identity, sexuality, cultural background and age as well as recruitment locations in order to represent young people visiting YHCs. A heterogeneous group of participants was sought in order to reflect the health perceptions of the diverse group of young people attending the YHCs. This group varies in age, background, social circumstances and health. This was of importance also because studies show that the YHCs are deemed less youth friendly by minority groups [26]. Recruitment was carried out among patients seeking treatment at the YHCs for a range of reasons, with most participants recruited either at a visit with a midwife or a social worker. This was done to make sure that patients seeking for a range of issues including mental health issues directly or indirectly, were included.

Young people who fulfilled the inclusion criteria and who showed interest in the study were given written and verbal information concerning the study by the staff at the YHC. The same staff also collected the young people’s contact information. They were then contacted by a member of the research group who further informed about the study and the larger research project, tasked with the development of an electronic patient reported outcome (ePRO), an instrument for measuring the health of patients to be used in YHCs, with the possibility to ask questions. Upon agreeing to participate, an appointment was booked for an interview. Interviews were held at the YHCs which they had visited or at nearby hospital facilities. None of the participants had any prior relationship with the interviewers and all were informed that the interviewers were researchers in the research project.

### Data collection

Interviews were carried out between June and November 2018 and during March 2023. Inclusion in 2023 was done to capture a wider range of health perceptions, since perception following the COVID-19 pandemic might have shifted. Interviews were continued until the research group agreed that further interviews seemed to lead to no new information, and when the interviews carried out in 2023 resulted in no new subcategories in the analysis, data collection was terminated. At the start of the interview, participants received written information concerning the study and written informed consent forms which were signed prior to commencing the interviews. A semi-structured interview guide was used for the interviews, which were carried out by two of the authors (MA, PLV), one of whom is a male psychologist, and the other a female physiotherapist, both of whom were PhD students at the time and both of whom have extensive clinical experience with young patients. Only the interviewer and the participant was present during the interview, and only one interview was carried out with each participant. The interviews focused on the participants’ view of mental health, how they perceive mental health, how they take care of their mental health and how they would prefer to be approached in order to talk about their health. The interview also included questions about physical and sexual health, as well as questions concerning preferences in the development of an ePRO for use at YHCs (the analysis of which has been published [36]), see Appendix 1 for the full interview guide. The interviews varied in length between 20 and 60 min and were recorded digitally and later transcribed verbatim by MA and hired administrative staff. No notes were taken during the recording.

### Data analyses

Inductive content analysis [32, 33] was used to analyse the transcribed interviews. The interviews were repeatedly read through and compared to recordings. Three separate interviews chosen at random were then independently split into meaning units relevant to the research question and coded by two of the authors (MA and ÅR) to check for consistency in coding. After having reached consensus concerning codes, one author coded the remaining interviews (MA). MA and ÅR then sorted codes from two more interviews, again chosen at random, into themes, categories and subcategories collaboratively, after which MA sorted codes from the remaining interviews with continuous feedback from ÅR. When consensus was reached between MA and ÅR, the remaining co-authors were invited to validate the analysis and to discuss themes and categories. All of the remaining authors participating in the analysis of data have long experience of health care and healthcare research (ÅR, EO, AB, LR) and specifically qualitative research (ÅR, AB, LR). The abstraction process was deemed to be complete when consensus between all researchers had been achieved. An example of the abstraction process from meaning unit to theme can be found in Table 2.

**Table 2:** Example of analysis from meaning unit to theme

Meaning Unit	Code	Sub-category	Category	Theme
But it’s easier if you’re not alone, like, if you talk to someone, and talk with someone about it, that’s what I’d think.	Easier to take care of mental health if you talk to someone about it.	Not being lonely.	Social influences	Mental health is helped and hindered by the surroundings
And at the same time I have, I’d say, quite a lot of anxiety and worry, and a hard time, like getting life sorted.	Has a lot of angst and a hard time getting life sorted.	Feeling good or bad can be lots of different things	How good and bad mental health works	Mental health is difficult to understand and difficult to achieve

## Results

Analysis of the data resulted in two themes, “Mental health is helped and hindered by the surroundings” and “Mental health is difficult to understand and difficult to achieve”. These, in turn, are divided into five different categories and numerous subcategories, see Table 3 for details.

### Theme 1: mental health is helped and hindered by the surroundings

The participants expressed that their social surroundings played a central role in their mental health. Their social surroundings appeared to be the arena in which their mental health primarily played out. However, the social surroundings could both facilitate and help with mental health status as well as cause tremendous distress, which led to vulnerability and lack of control. The participants also appeared to be highly aware of the healthcare system as a place to go for help when it came to mental health, but experiences of accessing it for treatment varied, and certain prerequisites needed to be met in order for young people to feel that they could make use of the health services provided. This theme consists of 3 categories.

#### Category: social influences

The social environments of the participants were of huge importance to the mental health of young people according to the interview data. It was however clear that all of these different institutions may have both a positive and a negative effect on mental health. The family as a support structure, for example, was by some felt to be generally positive, with one participant explaining that mental health is achieved through “getting to be with my family and spending time, like” (IP 11). Others, however, had a largely negative experience of turning to their family members for support concerning mental health, because “you feel like ‘Oh no, now I’m going to let my parents down’, and that’s a big issue” (IP 7). Similarly, friends and partners could be both good and bad support, and many felt that the most natural place to go when it came to mental health was peers, while others expressed that “the most important thing is your company… I think the worst things happen there, actually” (IP 13).

**Table 3:** Themes, categories and sub-categories resulting from the analysis

Theme	Category	Sub-category
Mental health is helped and hindered by the surroundings	Social influences	The family as support
		Friends and partners can be both good and bad to have around
		School is both important and a cause of stress
		Society as a whole should be better at helping
		The internet is supportive, but can also make things worse
		Not being lonely
	The healthcare system and its function	Some people feel helped, others do not
		You know you’re sick when the healthcare services react
		You have to ask us how we’re feeling!
	What is needed to be able to get help	What others think about mental health may give feelings that are difficult to handle
		Meeting a real person and feeling safe
		Getting to choose for yourself
		It’s not supposed to be so difficult
		We need to get info
		We’re different individuals with different needs
Mental health is difficult to understand and difficult to achieve	How good and bad mental health works	Feeling good or bad can be lots of different things
		Mental health is our main problem!
		Different kinds of health depend on each other
		Hard to know and show how you’re feeling
		You know how you’re feeling depending on how you’re doing socialy
	Being able to manage your health	Taking care of yourself
		How I’m feeling is my identity

In the interviews, young people expressed that school was both considered important, but also their biggest cause of stress. School was also mentioned as an important place for outreach to young people and a good place to notice poor mental health, while at the same time being perceived as not being very good at doing it. School health services, for example, were perceived as a valuable possible resource, but as IP 6 put it: “I’m thinking that we’ve done check-ups with the school nurses, and they don’t talk so much about mental health. They focus mostly on physical health. We could use some more there”.

Similarly, the young people interviewed also viewed different societal institutions, such as the social services, different religious institutions, and non-government organisations as both helpful and as a hindrance to achieve and maintain mental health. Particularly the social services were viewed negatively, and their involvement was said to have a negative impact on mental health. It was also mentioned that the responsibility for helping young people lay not with specific institutions, but rather with society as a whole. In the interviews, it was mentioned that it would be helpful if the topic of mental health was more broadly discussed in general in order to aid youth people with their mental health problems, for example “… so for my part I think the healthcare system in itself will have a hard time figuring out why we’re feeling so bad, it’s more a question for society, in general, I should think.” (IP 14).

The internet was mentioned as a supportive resource for support and information, particularly for LGBTQIA + youth, but also as a potential problem for young people as some online arenas tend to encourage poor health rather than help with feeling better, as attested by IP 8: “You just search for something, and you just get on to something, like… Now I’m going onto [a site about] eating disorders again, but people are just writing ‘does anyone want to be my friend so that we can help each other starve’ and stuff”.

Finally, the interview material also seemed to indicate that while social support was seen as contributing to both good and bad mental health, not being lonely was perceived as particularly important. The young people who were interviewed felt loneliness was to be avoided in order to stay healthy, and a common action taken to take care of mental health was to make sure to spend time with other people. When asked what gives poor mental health, IP 10 says ”not being involved in your own life, not getting a social life”, while IP 7 added that “when I’ve felt that I’ve got support, then I’ve, like, it [mental health] hasn’t been a hindrance in my life”.

#### Category: the healthcare system and its function

The participants in the interviews viewed the healthcare system as important to their mental health needs, although opinions were split on the degree to which the current healthcare system meets their needs as it is currently structured. The healthcare system also had other important functions, apart from administering healthcare, such as conferring legitimacy to your experience when you feel bad and asking young people how they are feeling.

In the analyses, it was clear that some participants felt helped by the healthcare system, while others did not. To illustrate the divide, IP 5, in response to a question concerning what they would do if they needed help with mental health issues, responded “Then I’d probably look for help via the youth health clinic and look for some sort of psychologist or social worker”, while IP 17, who mentioned having had long experience of the psychiatric services, stated “Like, I’m going to be completely honest, I think the healthcare system here in [town] is really sucky when it comes to mental health”. Specific complaints directed at the general health services also included long waiting times to get help and lack of knowledge in LGBTQIA+-relevant issues, whiles the YHCs were viewed more favourably. It was also mentioned that staff in the healthcare services have good intentions but are not able to reach positive results in treatment.

A separate function fulfilled by the healthcare system was that of adding legitimacy to experiences of poor health. The young people interviewed mentioned feeling validated in feeling sick when the healthcare services reacted, making receiving care from the healthcare system a way of defining what constitutes ill health as opposed to just feeling bad. Attention from the healthcare system could also lead to taking your problems more seriously. Unfortunately, this also means that long waiting times within the system led to feelings of insecurity, with one person interviewed expressing that ”if you don’t even know, you can’t even be certain if I really have these problems or if it’s something that I’ve had for a long time that’s just been imagined” (IP2).

In the interviews, it was also mentioned that young people rarely volunteer to adults in general, or the healthcare system in particular, that they are suffering from poor mental health. Young people said they needed healthcare staff to ask how they’re feeling to be able to open up about their healthcare needs. This was presented as a necessity for accessing help. In the interviews, it was clear that being asked about how you were feeling may cause young people to stop and think about how they’re doing, with one saying that “a general question, ‘how are you feeling’, like, is a good start to a conversation… Because then maybe that gets you thinking, how am I doing today, and how have I been feeling recently?” (IP 9). There was, however, no consensus about what questions to ask or how to ask them, even though young people seemed positive to being asked questions about serious signs of poor mental health, and of suicidal ideation and self-harm specifically.

#### Category: what is needed to be able to get help

From the interview data, it was clear that participants felt the need for certain prerequisites to facilitate their help-seeking from the healthcare system, but also that there were certain obstacles to seeking help that needed to be surpassed. Several factors that were perceived either to make contact easier, or to be necessary to be able to seek help, were brought up, as well as pitfalls to opening up about mental health issues.

In the interviews, it was expressed that what others think about mental health may result in feelings such as guilt, shame and anxiety that are difficult to handle when it comes to help-seeking, and that may form a significant barrier to help-seeking. IP 15 said that ”I always say I’m feeling fine even though I’m not” due to complicated feelings around other people knowing that they were not doing well. Some went so far as to put specific words to the feelings, for example ”… maybe they don’t want to say so themselves, but they know… they don’t really want to tell anyone else because they feel the shame is too great and so on” (IP 10).

To facilitate help-seeking that may feel difficult, participants expressed specific needs to be able to seek help. They reported a need to meet a real person and to feel safe during visits within healthcare, which entails a need for staff members that give a positive, accepting impression to the patients. Sometimes this meant a face-to-face, personal appointment with a member of staff who could “show that, oh my God, this person actually cares about how I’m feeling, that can really be worth a lot” (IP 4). Some, however, expressed a preference for digital appointments, since that felt safer. Emphasis in the interviews was also placed on getting to choose for yourself how and when to receive care and on the importance of integrity concerning young people’s healthcare choices. This included being able to access healthcare without parents or other adults necessarily finding out about this. In IP 15s words: “I know there’s secrecy and all that but preferably it should say somewhere that ‘this will not be shown to anyone else’ because then you feel even safer because you don’t want everyone to know about your life in that way.”

Participants interviewed also expressed that it should not be difficult to get help from healthcare services. This included booking appointments that fit with their schedules and convenient locations for the clinics. They also expressed that talking to someone about their mental health should feel easy and not like a “big deal”, “So that you should feel like it’s easy, like, ‘I’ve got someone to talk to there” (IP 9). They also mentioned a need to get adequate information concerning their healthcare choices, as expressed by IP 2: “And that’s the thing, concerning that you want your information to get out, then it has to be so broad that it gets out there… And via schools too, schools play an important role to… Like get [the information] to where the kids are.”

Finally, participants in the interviews expressed that they are different individuals with different needs, which they required that the healthcare system take into account. They expressed varying preferences when it came to help-seeking, and LGBTQIA + youth in particular felt that the healthcare services were inadequate for their needs. It was often emphasised that mental health means different things to different people, and that diagnostic labels may be unhelpfully narrow. According to IP 21, “…it’s important [for staff] to be pretty open, not to jump to conclusions, since everyone has different experiences and come with different baggage, like…”. It was clear that young people in the interviews had a high need of feeling understood by their surroundings in order to be able to ask for help.

### Theme 2: mental health is difficult to understand and difficult to achieve

Mental health is a complicated subject, something which was apparent to the young people interviewed in the present study. Mental health was often, by the participants, perceived as something unstable, a goal that one had to constantly work hard at without necessarily knowing what it was one had to do. It was also something that participants had a hard time defining. This theme is split into 2 categories.

#### Category: how good and bad mental health works

Participants in the interviews sometimes struggled to define what they meant by mental health but could at other times be quite specific about what good and bad mental health was. Mental health was also hard to communicate about and was connected to both physical health and social circumstances.

The interviews contained comments that illustrated that feeling good and bad could mean lots of different things. Poor mental health was expressed to be equated with or caused by inner states like feeling stressed, anxious, depressed and having low self-esteem while good mental health was associated with happiness and the absence of sadness, and also concerned with how you handle situations. Mental health, in fact, was “about, like, how you take in things with your brain” (IP 9). Comments on mental health being difficult to define were common, while specific factors such as time of day or year were thought to influence mental health. They described that different kinds of health depended on each other, with IP 5, for example, saying that she “… can get, if I’m not feeling good mentally, I’ll feel it physically as well… I don’t get headaches, like, but I usually tense up quite a lot, in my stomach, I think, and in the body, I get pretty tense…”. They were clear, however, that mental health was the main problem facing young people, and that it constituted their major health concern, with one subject saying “Stress. Anxiety. Worry. Yes, amongst other things, there’s some other stuff as well, but that’s the most fundamental thing in society today” (IP 13). When asked about “health” in general, some reverted to talking only about mental health.

Young people described in the interviews that it was hard to know and show how you’re feeling. The difficulty lay both in knowing your mental state, but also in conveying to others how you were feeling. This difficulty was caused by different people having different interpretations of mental health, but also by the fact that you couldn’t necessarily tell how someone was feeling from the outside. IP 14 stated that “… I know myself what it’s like, maybe you don’t want to seem to be feeling better than you actually are, but you can get, like ‘There’s always someone worse off than you are’”, which also highlights the risk of being misunderstood or having your suffering diminished by others.

Finally, participants reported knowing how they were feeling depending on how they were doing socially. Being able to perform in social situations, such as your family context, amongst friends and in school, was part of the definition of having mental health. IP 18 explained, concerning how to know that they are suffering mentally, that “… I become more withdrawn from those close to me than, like, I keep a bit more to myself and stuff, and don’t do as much stuff as before”. Hence, someone not interacting with peers or not performing in school was a way of knowing that they were not feeling well mentally.

#### Category: being able to manage your health

Young people in the interviews seemed to perceive taking care of your mental health as an ongoing undertaking, where you continuously had to act in order to stay healthy. Mental health was also seen as an important part of your identity.

When young people in the interviews talked about taking care of themselves, they often mentioned doing things like meeting and talking to friends or exercising, but many other activities were also mentioned. One went so far as to say that “… I want to try to avoid the words ‘health, sick’, because I think health is something you do and not something you are” (IP 10). Having mental health was perceived as an ongoing project. It was also clear that staying healthy was something you could learn to get better at. Particularly participants who have experience of poor mental health said that how they were feeling was a part of their identity, and that feeling good could feel frightening. This is expressed by IP 8 as follows: “If you’ve felt bad for such a long time then you don’t dare feel good again, because that can then disappear. In that case you’d rather feel bad all the time”.

## Discussion

This interview study gives many insights into how young people visiting YHCs perceive the concept of mental health and how they try to achieve it. From the inductive analysis of the interviews, two themes emerged; (1) Mental health is helped and hindered by the surroundings and (2) Mental health is difficult to understand and difficult to achieve. Both themes indicated uncertainty in having mental health from a young person’s point of view. Theme 1 shows that mental health is perceived as heavily influenced by social surroundings, which are often outside one’s control, while Theme 2 shows that mental health entails hard, continuous work, even though one might not know what the end goal is. Taken together, this entails that young people may find conversations about mental health intimidating, while at the same time expressing a need to be asked how they’re doing in order to be able to talk about how they are feeling. At the same time, young people interviewed underline that they need adults in general, and health care professionals in particular, to ask about their mental health, as they will not give this information without prompting. These insights should be kept in mind when approaching young people concerning mental health.

The centrality of the social surroundings for young people’s mental health is in the present study brought to the forefront. The potential positive and negative impact of friends, family, and schooling is at the core of how young people describe health. This is echoed in some previous findings concerning how young people perceive health generally and mental health particularly [27–30], but is further emphasised in the present material. These findings also mirror findings concerning the importance of peers and family to mental health in young people [37, 38], and are also in tune with the centrality of social stimuli to the adolescent developmental phase [3, 4]. The findings in the present study also show that the young people interviewed are aware of the connection between social circumstances and mental health. This, in turn, holds important implications for both assessments of how young people are feeling, which may achieve increased accuracy by including a social perspective, and treatment of poor mental health. It also implies that mental health should be an important focus for school health services, a suggestion that is brought up in the interviews. However, it is, as stated above, important to keep in mind that young people may feel uncomfortable discussing how social circumstances affect their health, and the circumstances of the individual need to be taken into account.

The need for an individualised, person-centred approach for young people in the healthcare system is also brought up as a prerequisite for young people being able to, or rather being motivated to, access healthcare. Person-centred healthcare in adult patient populations has been found to increase self-efficacy and satisfaction with care [39], amongst other findings, and is also a government priority in Sweden [40]. That the young people interviewed in the present study clearly state a need for a person-centred approach underlines the need for further effort from the YHCs specifically, but the healthcare establishment in general, to expand research and healthcare efforts towards person-centred care for this particular group.

Several of the criteria in the guidelines set up by the WHO for YFHSs [18, 20], particularly the criteria of accessibility (free healthcare and flexible hours/modalities for contact), acceptability (individualised care, trust and safety, right to secrecy) and appropriateness (seeing the whole individual), are also highlighted as particularly important for healthcare services to attract patients. On one hand this seems reasonable, since the young people interviewed were all patients at YHCs, clinics that seem to approximate the YFHS guidelines. Previous studies have also shown that YHCs are perceived as youth-friendly by young people [23, 25]. However, in [25], for example, the semi-structured interview guides were based on the central domains of YFHS, while the present study had a more general aim and structure of the interview guide. The fact that naïve subjects, who are unaware of the WHO guidelines, still bring up these criteria for being able to access healthcare spontaneously may be interpreted as an indicator of the content validity of the WHO guidelines themselves.

Young people interviewed on the one hand seemed to contrast the YHCs with conventional health care, but on the other hand had uniform expectations on “health care services”. In the interviews, two questions enquired about how “health services” should approach young people. The way the term “health service” was used in the interviews can apply to all health care, including both “regular” health services, such as primary care or psychiatric care, and the YHCs. In the interviews, young people tended to separate “regular” health services and YHCs and view them as different entities in response to questions concerning “health services”. The “regular” health care services, particularly the psychiatric services, were sometimes brought up as an example of negative experiences with seeking help, despite not being the focus of the interviews, whiles the YHCs overall received positive reviews. This is not surprising since the present study used only participants who for different reasons choose to seek out the YHCs, but it is a clear indicator that “regular” health services at least by some are not viewed as “youth-friendly”. It also serves as a reminder of the importance of the guidelines to facilitate health care access for the group.

Finally, it is important to note that mental health and mental health problems to the young people interviewed seem to be the most important aspect of their health. The young people echoed findings in current research [1, 5] and referred to mental health as the biggest health issue facing young people today. In some of the interviews “poor health” even seems to be used synonymously with “poor mental health”. Although not surprising, since young people are generally physically healthy [1, 2], it is also worth noting that mental health, and particularly poor mental health, seems to be an important part of young people’s self-image, even to the extent that poor mental health may feel safe compared to striving to attain good mental health. Self-image has in numerous studies been found to have causal relationships with poor mental health [41], and studies have also found that young people who engage in behaviours such as non-suicidal self-injury sometimes do so to strengthen a sense of belonging to a peer group [42]. This finding may hold implications for treatment of mental health as motivation for improvement may be lacking if good mental health in itself is perceived as a frightening prospect. Further research may focus on how poor mental health forms part of how young people view themselves and if this in turn affects the recovery process.

The present study has certain limitations. The additional interviews performed in 2023 did not include any participants identifying as non-female, meaning that any possible difference in perceptions and needs between 2018 and 2023 in male and youth with other gender identities at the YHCs may not be captured in the study. While the gender distribution as well as other background factors in the present sample reflects the population who attend YHCs, findings may not be transferable to adolescents and young adults who do not seek treatment at YHCs. Also, in order to respect the YFHS guideline of a right to secrecy, no participants who would have required parental consent were recruited for the present study. This entails that the findings may not reflect the views of the very youngest people attending the YHCs.

Interviews were carried out by two separate interviewers (MA and PVL) and codes from both interviewers went into forming all categories and themes. No differences in themes or categories were found between interviewers, indicating that the results of the interviews carried out by both interviewers are substantially the same. The second interviewer read the interviews carried out by the first interviewer before performing the additional interviews which may strengthen dependability. Conversely, it may also pose a threat to dependability, since the second interviewer may have been influenced by the first set of interviews. The interview guide used for the semi-structured interviews was not pilot tested prior to the interviews, implicating a risk that the questions asked were insufficient to fulfil the stated aim of the study. However, the interview guide consistently asked participants questions in an open manner, as well as continuously asking if there was anything further they’d like to ask, which may lessen this risk. Also, while the participants in the study as stated above reflect the population at YHCs, no data was kept on who was approached by staff and asked to participate, which means there is no way for the present study to account for who refused to participate in the study or why. Finally, the participants were not shown the transcripts of the interviews. While the transcripts are verbatim from the recordings, participants have thus not had the opportunity to correct any misunderstandings arising in the interviews. Nor were participants asked to give feedback on findings from the analysis, which also entails a risk of lack of depth in the analysis.

In order to enhance trustworthiness and improve the credibility of the data analysis, both coding and analysis were validated by different members of the research group. The first author, MA, has many years of experience working as a psychologist at a YHC, which gives unique insight into the topic studied. However, this experience with working in a YHC may also entail bias concerning the patient group or how they perceive mental health. No other authors involved in the analysis process had specific experiences from YHCs, ameliorating any bias.

## Conclusion

The present study sheds light on how young people perceive the concept of mental health and how to handle and achieve mental health, with particular emphasis being placed on the social element of mental health, on specific needs for accessing help, and on the centrality of mental health to young people. It also holds important implications for the need of focusing on person-centred and youth-friendly health care options for the age group, who may otherwise fail to receive adequate care. Society as a whole, but also specific stakeholders such as the school system, also need to focus more on mental health and enquiry concerning mental health in order to aid young people who are suffering from poor mental health in speaking of their problems. While the present study provides important insight into how young people perceive the concept of mental health and may help to improve how the healthcare services approach young people concerning their mental health, further studies drawing subjects from a non-clinical setting may expand on our knowledge of what mental health means to young people. Also, the present study has only involved young people as objects of study. By including adolescents and young people more intimately as stakeholders in research, valuable perspectives and further insights may well be acquired.

### Supplementary Information

**Supplementary Material 1.**

## Data Availability

No datasets were generated or analysed during the current study.

## Abbreviations

WHO: World Health Organization
YFHS: Youth-friendly Health Service
YHC: Youth health clinic
ePRO: Electronic Patient Reported Outcome
