# Supplementary material for: “It’s about how you take in things with your brain” - young people’s perspectives on mental health and help seeking: an interview study
Source: BMC Public Health. 2024 Apr 20;24:1095. doi: 10.1186/s12889-024-18617-4 (PMC11031856; doi:10.1186/s12889-024-18617-4)
Supplement: Supplementary file 1 — Supplementary Material 1. [file 12889_2024_18617_MOESM1_ESM.docx]

**INTERVIEW GUIDE**

Welcome!

**PURPOSE**: The purpose of the study is:

1. To get a better understanding of what young people experience and perceive physical, mental and sexual health to be, what they would like help with, and what expectations they have on YHCs when they seek help with their health.
2. We’re also wondering what young people think about developing an application that captures the needs of young people, in order to give YHCs better foundations for their assessment of how young people are feeling and what kind of help you need.

Give information concerning the interview process:

- Takes up to 60 minutes.
- You can terminate the interview whenever you choose without it in any way effecting your continued contact with the YHCs.
- Confidential, your answers will by no means be connected to you as a person, no-one will be able to find out that you have participated or what you have answered.
- Would you require any travel compensation?
- Would you be willing to sign the consent form to participate in the study?

**START OF THE INTERVIEW**

I will start by asking a few things about you, and will then ask further questions in three areas:

**Health**

**Support and expectations**

**Application**

I would like you to speak as freely as possible, even if we start getting slightly off topic. If we get too far away from the topic of the question, I will try to restrict the conversation and get back on topic. Would that be okay?

**SUBJECT 1**

**Health**

*Main question 1:*Could you tell me what “health” means to you?

*Subquestions:*

Can you mention a few things that have to do with physical health/poor physical health?

Please tell me what you usually do if you need help with something that concerns physical health?

How do you take care of your health? Food, exercise, sitting still, sleep, stress, sexually?

Can you mention a few things that have to do with mental health/poor mental health – how you are feeling inside?

Please tell me what you usually do if you feel bad inside, like sad or down?

Can you mention a few things that have to do with sexual health/poor sexual health?

Please tell me what you usually do if you need help with something that concerns sexual health?

*Main question 2:*How come you are getting in touch with the YHCs on this occasion?

*Subquestions:*

How do you think the reason for you coming to the YHC on this occasion effects your health in general? (mentally/physically/sleep/appetite/sexually and so forth)

**SUBJECT 2**

**Support and expectations**

*Main question 1:*

What is the reason for you seeking help at the YHC?

*Subquestions:*Have you ever come to the YHC for help previously? Could you please tell me why and what kind of help you received at that point?

Main question 2:

What kind of help are you hoping the YHC can give you?

*Subquestions:*

Please tell me about what kind of services the YHCs can provide?
Do you think there’s anything missing, that you would like the YHCs to provide?

Have you turned anywhere else looking for help? If so, where? (school health, teachers/other adult at school, child primary care services, child psychiatric services, NGOs?)

What was the reason you sought help at that point?

**Applications**

*Main question 1:*

What do you think is important for health services to know about how best to find out how young people are feeling?

*Subquestions:*

What would be important for health services to consider in order to be able to provide the care that young people want?

What improvements do you think the YHC could make so that young people can be cared for in the best way by the YHC?

*Main question 2:*

What do you think a digital application should contain in order to capture how you are feeling?

*Main question 3:*

How would you feel about being asked to use an application about your health before your first visit to the YHC?

*Subquestions:*

In what way would it be positive to use an application in order to communicate how you are feeling?

In what way would it be negative to use an application in order to communicate how you are feeling?

Do you have any opinion on whether to use your own phone or a digital tablet to use the digital application?

Are there any benefits to using the application when already at the YHC? Are there any downsides to using the application on site?

*Main question 4:*

How many questions would you feel okay answering in an application? How long should filling out the application take?

*Subquestions:*

What kind of questions should definitely NOT be in the application? What kinds of questions should DEFINITELY be in the application?

**IN CLOSING**

1. We are now getting to the end of this interview. I’m going to make a brief summary of what we have been talking about…

2. … Is there anything you think I should have asked about that we didn’t talk about? Could we talk about that now instead?

3. Is there anything else you would like to add?

4. How have you felt talking to me about these questions?

5. Would it be possible to meet again so that we can give you feedback concerning the outcome of the interviews, and so that you can comment on whether we have been able to accurately capture things you view as important to get right when young people seek help?

6. Would you consider being part of further development of the application to for example look at questions included to see if they make sense? If this becomes relevant, we’ll contact you.

7. We may also need help in developing the application. Would you be interested in helping us do that? If this becomes relevant, we’ll contact you.

Thank you for your help!
